# Effect of mineralocorticoid receptor antagonists on proteinuria and progression of chronic kidney disease: a systematic review and meta-analysis

**DOI:** 10.1186/s12882-016-0337-0

**Published:** 2016-09-08

**Authors:** Gemma Currie, Alison H. M. Taylor, Toshiro Fujita, Hiroshi Ohtsu, Morten Lindhardt, Peter Rossing, Lene Boesby, Nicola C. Edwards, Charles J. Ferro, Jonathan N. Townend, Anton H. van den Meiracker, Mohammad G. Saklayen, Sonia Oveisi, Alan G. Jardine, Christian Delles, David J. Preiss, Patrick B. Mark

**Affiliations:** 1 Institute of Cardiovascular and Medical Sciences, British Heart Foundation Glasgow Cardiovascular Research Center, 126 University Place, Glasgow, UK; 2 Division of Clinical Epigenetics, Research Center for Advanced Science and Technology, The University of Tokyo, Tokyo, Japan; 3 Department of Clinical Study and Informatics, Center for Clinical Sciences, National Center for Global Health and Medicine, Tokyo, Japan; 4 Steno Diabetes Center, Niels Steensens Vej, Gentofte, Denmark; 5 Health, Aarhus University, Aarhus, Denmark; 6 NNF Centre for Basic Metabolic Research, University of Copenhagen, Copenhagen, Denmark; 7 Department of Nephrology, Herlev Hospital, University of Copenhagen, Herlev, Denmark; 8 Departments of Cardiology and Nephrology, University Hospital Birmingham and School of Clinical and Experimental Medicine, University of Birmingham, Birmingham, UK; 9 Department of Internal Medicine and Pharmacy, Erasmus MC, Rotterdam, The Netherlands; 10 VA Medical Center, 4100 West Third St, Dayton, OH 45428 USA; 11 Metabolic Diseases Research Center, Qazvin University of Medical Sciences, Qazvin, Iran; 12 Clinical Trial Service Unit and Epidemiological Studies Unit, University of Oxford, Oxford, UK

## Abstract

**Background:**

Hypertension and proteinuria are critically involved in the progression of chronic kidney disease. Despite treatment with renin angiotensin system inhibition, kidney function declines in many patients. Aldosterone excess is a risk factor for progression of kidney disease. Hyperkalaemia is a concern with the use of mineralocorticoid receptor antagonists. We aimed to determine whether the renal protective benefits of mineralocorticoid antagonists outweigh the risk of hyperkalaemia associated with this treatment in patients with chronic kidney disease.

**Methods:**

We conducted a meta-analysis investigating renoprotective effects and risk of hyperkalaemia in trials of mineralocorticoid receptor antagonists in chronic kidney disease. Trials were identified from MEDLINE (1966–2014), EMBASE (1947–2014) and the Cochrane Clinical Trials Database. Unpublished summary data were obtained from investigators. We included randomised controlled trials, and the first period of randomised cross over trials lasting ≥4 weeks in adults.

**Results:**

Nineteen trials (21 study groups, 1 646 patients) were included. In random effects meta-analysis, addition of mineralocorticoid receptor antagonists to renin angiotensin system inhibition resulted in a reduction from baseline in systolic blood pressure (−5.7 [−9.0, −2.3] mmHg), diastolic blood pressure (−1.7 [−3.4, −0.1] mmHg) and glomerular filtration rate (−3.2 [−5.4, −1.0] mL/min/1.73 m^2^). Mineralocorticoid receptor antagonism reduced weighted mean protein/albumin excretion by 38.7 % but with a threefold higher relative risk of withdrawing from the trial due to hyperkalaemia (3.21, [1.19, 8.71]). Death, cardiovascular events and hard renal end points were not reported in sufficient numbers to analyse.

**Conclusions:**

Mineralocorticoid receptor antagonism reduces blood pressure and urinary protein/albumin excretion with a quantifiable risk of hyperkalaemia above predefined study upper limit.

**Electronic supplementary material:**

The online version of this article (doi:10.1186/s12882-016-0337-0) contains supplementary material, which is available to authorized users.

## Background

Chronic kidney disease (CKD) is associated with risk of premature cardiovascular (CV) disease and death [1–6]. Hypertension (HTN) is the major modifiable risk factor for CKD progression and is associated with development of left ventricular hypertrophy (LVH) and proteinuria, both predictors of CV mortality [3, 7]. In CKD patients with proteinuria and/or HTN renin angiotensin system (RAS) inhibitors are commonly prescribed as these agents have been shown to reduce proteinuria and delay CKD progression through a combination of BP dependent and independent mechanisms. Despite this, patients still progress to end stage renal disease (ESRD) or die from CV events [8–14].

There is renewed interest in aldosterone as a mediator of CV and renal disease, beyond its BP effect resulting in an enthusiasm for using mineralocorticoid receptor antagonists (MRA) to minimised proteinuria and delay CKD progression. A 2009 meta-analysis, updated in 2014 demonstrated that addition of MRA to RAS blockade reduced BP and proteinuria in CKD [15, 16]. The beneficial effects on outcomes were confounded by increased risk of hyperkalaemia, a factor limiting MRA prescribing in CKD [17–19]. Similar findings were described in a more recent meta-analysis on the cardiovascular actions of MRAs in CKD [20]. However, the conclusions of these analyses are drawn from mainly published proteinuria data only, derived by consolidating disparate urinary protein excretion measures used in different trials (variably reported as protein or albumin excretion in spot samples or 24 h collections). Furthermore, in some studies in these meta-analyses, the MRA effect is impossible to dissociate from that of additional antihypertensives co-administered with MRA in the intervention arm.

In the past 6 years several studies of effects of selective and non-selective MRAs in CKD have been published [21–34]. We performed an updated meta-analysis of this treatment strategy using summarised unpublished data where possible, as well as including data from 3 studies which were not considered in the previous publication [28, 30, 34]. This is particularly relevant as one of these studies focussed on patients with CKD stage 3–4 [30] an area where evidence for use of this strategy is lacking, and also because the resultant number of participants included exceeds that of the previous publications. We focused on change in urinary protein/albumin excretion, progression of CKD and risk of hyperkalaemia whilst additionally collecting hard clinical endpoints where these data were available. Our aim was to determine whether renoprotective benefits of MRAs outweigh risk of hyperkalaemia associated with this treatment.

## Methods

Literature search was performed independently by two authors (GC, AT) using PubMed (1966 - 1st Dec 2014), EMBASE (1947 - 1st Dec 2014) and the Cochrane Clinical Trials Database. Search strategy is shown in Appendix 1 (see Additional file 1).

### Trial type

We analysed randomised controlled trials in humans published in English of both selective and non-selective MRAs performed in CKD stage 1–5 where MRA was compared to placebo or open label trials where MRA was additional therapy compared to the non-MRA arm. Trials were eligible if MRA was used alone or combined with ACE-I alone, ARB alone, or both ACE-I and ARB, performed in CKD patients or for prevention of CKD progression. The first period of randomised crossover trials was also considered eligible. Trials directly comparing MRA to other antihypertensive agents were excluded.

### Participants

Trials enrolling patients with CKD stages 1–5 not requiring RRT, with albuminuria or proteinuria were included [11]. Our search included haemodialysis, peritoneal dialysis and renal transplantation ensure all appropriate trials were identified but RRT studies were excluded from the main analysis to minimise the confounding effect of dialysis on blood pressure and potassium.

### Interventions

Trials using selective and non-selective MRAs with placebo, ACE-I, ARB or both were included. Minimum trial duration was 4 weeks.

### Outcome measures

Analysis plan included effects of MRAs on the following measures:

- End of treatment urinary albumin or protein excretion (24-h proteinuria or albuminuria, or urinary protein ratio or albumin creatinine ratio)
- End of treatment renal function: serum creatinine (μmol/L); eGFR (ml/min/1.73 m^2^); creatinine clearance (ml/min); doubling of serum creatinine; need for RRT. When several measures of kidney function were available this was meta-analysed following the hierarchy- isotopic GFR, creatinine clearance from 24 h urine collection, eGFR (MDRD or CKD-EPI formulae), formula estimated creatinine clearance (Cockcroft-Gault)
- End of treatment SBP and DBP (mmHg)
- Hyperkalaemia (serum potassium above trial protocol limit)
- Death, need for RRT, cardiovascular events.

### Data collection

The search strategy in Additional file 1: Appendix 1 identified titles and abstracts. Both reviewers assessed titles and abstracts independently, discarding those not meeting inclusion criteria. Full texts of remaining trials were independently assessed. A third author (PM) settled discrepancies. Data extraction was performed using specific extraction forms (Appendix 2, see Additional file 1). Original authors were contacted tor further information.

### Assessment of risk of bias

Two independent reviewers (GC, AT) assessed trial quality using the Cochrane Collaboration risk of bias assessment tool [35]. Items assessed were: adequate sequence generation; allocation concealment; blinding of participants, trial personnel and outcome assessors; reporting of incomplete outcome data; suggestion of selective outcome reporting and intention to treat analysis (Additional file 1: Table S1).

### Statistical analysis

Random effects meta-analysis was performed for continuous and categorical outcomes. For continuous outcomes, weighted mean differences were performed using two approaches: in analyses of continuous data, final visit results were compared for treatment and control arms after verifying by meta-analysis that baseline data for the relevant outcome were no different between trial arms; second, where sufficient data were available, change in weighted mean difference from baseline to final visit was calculated by meta-analysis. For categorical outcomes, we calculated risk ratios (RRs) as ratio of cumulative incidence and 95 % CIs from available data for trial participants at baseline and for those developing the outcome of interest. Random effects models were used as preferable approach to manage between-trial heterogeneity introduced by analysing differing trial populations. As standard deviations were unavailable for all measures of change in albuminuria and proteinuria, percentage change was analysed using weighted means and weighted standard deviations across trials in exploratory analyses. Statistical heterogeneity across studies was quantified using I^2^ statistics, providing measure of proportion of overall variation attributable to between-trial heterogeneity, with *p* < 0.10 considered significant. We assessed publication bias with funnel plots and Egger tests, for the most commonly reported outcome - SBP. Analyses were conducted using Stata version 13 (StataCorp, College Station, Texas).

## Results

### Literature search and trial characteristics

#### Search results

**Fig. 1:**
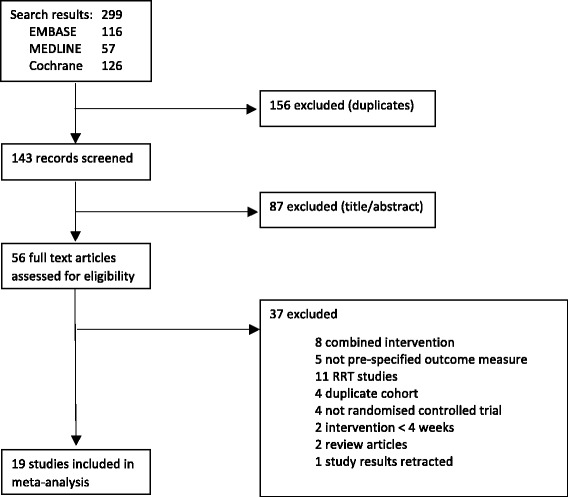
Study flow chart

Search of PubMed, EMBASE and the Cochrane database identified 299 citations. 243 were excluded (overlapping searches; non-randomised trials; trials evaluating interventions not included in this review) (Fig. 1). Full text assessment of 56 articles resulted in selection of 19 eligible trials including 1646 patients [22–31, 34, 36–43], more than were included in the previously published meta-analyses [15, 16, 20]. Authors were contacted for unpublished data; we obtained supplemental summarised results for ten trials [23, 26–30, 39, 40, 42, 43]. These were whole group mean and standard deviations pre- and post- intervention for the outcomes of interest, allowing a more complete and precise analysis.

One trial compared ACE-I and ARB (or placebo) and Spironolactone (or placebo), (four groups in total) [37] and another compared two doses of Eplerenone against placebo, (three groups in total) [38]. All comparable arms of both trials were included for analysis. Therefore, 19 trials of 21 study groups were included for analysis. Fourteen trials (889 patients) compared Spironolactone plus ACE-I or ARB with ACE-I or ARB alone; and 5 trials (757 patients) compared Eplerenone plus ACE-I or ARB to ACE-I or ARB alone. One trial focused on triple RAS blockade vs dual RAS blockade [41]. We did not identify any trials comparing MRA to placebo in the absence of ACE-I/ARB treatment.

#### Trial characteristics

Included were five randomised placebo controlled trials, seven randomised controlled trials (treatment compared to standard care) and seven randomised crossover trials. Six trials included patients with non-diabetic CKD, eight trials focused on diabetic nephropathy, 3 included both diabetic and non-diabetic CKD and 2 included patients with CKD and HTN.

Study duration was between 8 and 52 weeks. Sample size was small (*n* = 18 to 359). No trials were powered to measure mortality or long-term renal outcomes. Dose of Spironolactone was 25 mg/day in most trials, while two trials used 25–50 mg [24, 42]. Eplerenone dose ranged from 25 to 100 mg.

The primary endpoint in the majority of trials was change in urinary protein/albumin excretion, although there was significant heterogeneity in measures used. Trials reported change in urine protein:creatinine ratio (PCR); albumin:creatinine ratio (ACR) or 24-h urine protein/albumin excretion. In three trials change in protein/albumin excretion was a secondary outcome measure where BP, pulse wave velocity and left ventricular mass index (LVMI) respectively were primary endpoints [23, 24, 30].

In trials reporting estimated glomerular filtration rate (eGFR) calculation method included Modification of Diet in Renal Disease (MDRD); Cockcroft Gault creatinine clearance (CrCl) and Chronic Kidney Disease Epidemiology Collaboration (CKD-EPI) tools. In three trials GFR was measured by 51Cr-EDTA [26, 39, 40]. Characteristics of participants and interventions of included trials are listed in Table 1.

**Table 1:** Summary of populations and interventions in included studies. Data are mean ± SD or median (IQR)

Study	Kidney disease	No. of patients included	Intervention group	Control group	Co-intervention	Study duration	Baseline eGFR (ml/min/1.73 m^2^)	Endpoints
Abolghasmi 2011 [24]	CKD with resistant hypertension	41	Spironolactone 25–50 mg	Placebo	multi-drug regime including ACE-I+/−ARB	12 weeks	Not available	BP, potassium, creatinine, urinary sodium
Ando 2014 [28]	CKD with hypertension	314	Eplerenone 50 mg	Placebo	ACE-I+/−ARB of at least 8 weeks duration	1 year	Treatment 67.7 ± 14.3 Control 68.6 ± 13.6	UACR, creatinine, eGFR, urinary L-FABP, 24 h urinary sodium, incidence of cerebrovascular and cardiovascular events
Bianchi 2006 [36]	Non-diabetic CKD (idiopathic GN)	165	Spironolactone 25 mg	ACE-I+/−ARB	ACE-I+/−ARB	1 year	Treatment 62.4 ± 21.9 Control 62.2 ± 19.0	24 h urinary protein, BP, creatinine, eGFR potassium
Boesby 2011 [29] (XO)	Non-diabetic CKD	40	Eplerenone 25–50 mg	multi-drug regime including ACE-I+/−ARB	multi-drug regime including ACE-I+/−ARB	8 weeks	59 ± 26	24 h urinary albumin, BP, potassium, creatinine clearance
Boesby 2013 [30]	Diabetic and non-diabetic CKD	26	Eplerenone 25–50 mg	ACE-I+/−ARB	ACE-I+/−ARB	24 weeks	36 ± 10	cfPWV, AIx, AASI, 24 h urinary albumin
Chrysostomou 2006^a^ [37]	Diabetic and non-diabetic CKD	41	Spironolactone 25 mg	Placebo as ARB; Placebo as Spironolactone	ACE-I alone; ACE-I + ARB	3 months	Not available	24 h urinary protein, BP, creatinine, creatinine clearance, potassium
Edwards 2009 [23]	Non-diabetic CKD with no renovascular diagnosis	112	Spironolactone 25 mg	Placebo	ACE-I/ARB	36 weeks	Treatment 49 ± 12 Control 53 ± 11	LVMI, cfPWV, aortic distensibility, AIx, BP
Epstein 2006+ [38]	Diabetic nephropathy	359	Eplerenone 50 mg or 100 mg	Placebo	ACE-I	12 weeks	ACE ± EPL 50 73 (62.1–83.6) ACE ± EPL 100 75 (62.8–85.9) Control 74 (60.5–82.2)	UACR, potassium, BP, eGFR
Guney 2009 [25]	Non-diabetic CKD	24	Spironolactone 25 mg	ACE-I+/−ARB	ACE-I+/−ARB	6 months	Treatment 63.0 ± 22.71 Control 56.3 ± 35.6	UPCR, urinary TGF-β1, eGFR, creatinine, potassium, BP, aldosterone
Mehdi 2009 [22]	Diabetic nephropathy	81	Spironolactone 25 mg	Placebo or ARB	ACE-I	48 weeks	Not available	UACR, BP, creatinine clearance, potassium
Nielsen 2012 [26] (XO)	Diabetes with microalbuminuria	21	Spironolactone 25 mg	Placebo	ACE-I/ARB	60 days	Not available	24 h urinary albumin, BP, GFR, urinary L-FABP, urinary NGAL, urinary KIM-1
Rossing 2005 [39] (XO)	Diabetic nephropathy	20	Spironolactone 25 mg	Placebo	ACE-I+/−ARB	8 weeks	Not available	24 h urinary albumin, BP, GFR
Saklayen 2008 [43] (XO)	Diabetic nephropathy	24	Spironolactone 25–50 mg	Placebo	ACE-I/ARB	3 months	Treatment 61.9 ± 23.4 Control 54.4 ± 20.1	BP, creatinine, potassium, UPCR
Schjoedt 2005 [40] (XO)	Diabetic nephropathy	20	Spironolactone 25 mg	Placebo	ACE-I+/−ARB	2 months	Not available	24 h urinary albumin, BP, GFR
Tylicki 2008 [41] (XO)	Non-diabetic CKD	18	Spironolactone 25 mg	ACE-I + ARB	ACE-I + ARB	8 weeks	107.8 (93–140.9)	24 h urinary protein, BP, creatinine, potassium, PRA, urinary NAG, urinary PIIINP
Tylicki 2012 [31] (XO)	Non-diabetic CKD	18	Eplerenone 50 mg	ARB + Aliskiren	ARB	8 weeks	Not available	UACR, BP, creatinine clearance, potassium
van den Meiracker 2006 [42]	Diabetic nephropathy	53	Spironolactone 25–50 mg	Placebo	ACE-I/ARB	1 year	Treatment 93.1 ± 45 Control 66.3 ± 35.1	24 h urinary protein, BP, creatinine, eGFR, potassium
Wang 2013 [34]	Diabetic and non-diabetic CKD	208	Spironolactone 20 mg	multi-drug regime including ACE-I+/−ARB	multi-drug regime including ACE-I+/−ARB	16 weeks	Treatment 65.8 ± 22.2 Control 66.5 ± 24.3	24 h urinary protein, creatinine, potassium, eGFR, BP, aldosterone
Ziaee 2013 [27]	Diabetes with microalbuminuria	60	Spironolactone 25 mg	ACE-I	ACE-I	12 weeks	Treatment 79.8 ± 18 Control 82.5 ± 19.1	UACR, BP, potassium, eGFR

*UACR* urine albumin:creatinine ratio, *UPCR* urine protein:creatinine ratio, *ACE-I* angiotensin converting enzyme inhibitor, *ARB* angiotensin receptor blocker, *CKD* chronic kidney disease, *NG* glomerulonephritis, *L-FABP* liver-type fatty acid binding protein, *XO* crossover study design, *cfPWV* carotid-femoral pulse wave velocity, *AIx* augmentation index, *AASI* ambulatory arterial stiffness index, *LVMI* left ventricular mass index, *TGF-β1* transforming growth factor-β1, *NGAL* neutrophil gelatinase associated lipocalin, *KIM-1* kidney injury molecule-1, *PRA* plasma renin activity, *NAG* n-acetyl-β-D-glucosaminidase, *PIIINP* amino-terminal propeptide of type III procollagen, ^a^this study had 4 arms +this study had 3 arms

#### Trial quality

Trial quality assessed using the Cochrane Collaboration tool was variable (Additional file 1: Table S1). Sequence generation was adequately described in 9 (50 %) trials. Allocation concealment was adequate in 8 (44 %) trials. Participants and investigators were blinded in 12 (67 %) trials and intention to treat analysis was performed in 2 (11 %) trials. Dropouts were adequately accounted for in 16 (84 %) trials and did not differ between treatment and control groups.

### Trial outcomes

Baseline data from included studies is shown in Additional file 1: Table S2. Meta-analysis of baseline data for all outcomes of interest showed these were balanced, confirming that end-of-trial meta-analysis was appropriate (Additional file 1: Table S3).

#### Effect of treatment on blood pressure

**Fig. 2:**
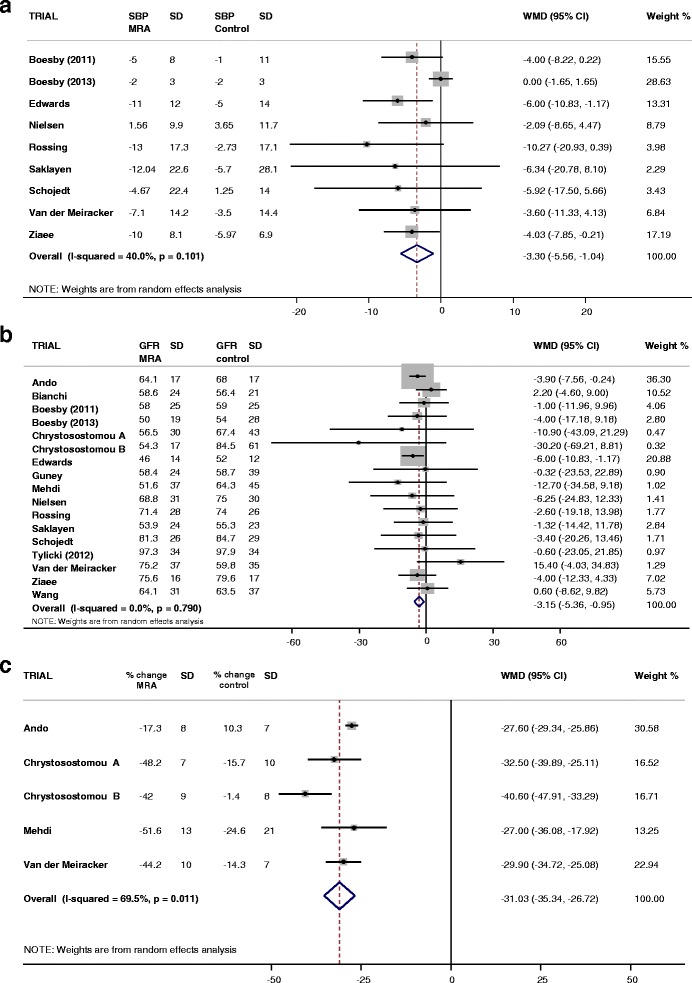
**a** Change in systolic blood pressure from baseline with addition of MRA to ACE-I and/or ARB compared to ACE-I and/or ARB alone. For participant numbers see Table 1. **b** Effect of the addition of MRA to ACE-I and/or ARB compared with ACE-I and/or ARB alone on end of treatment renal excretory function. For participant numbers see Table 1. **c** Percentage change from baseline of any measure of urinary protein/albumin excretion with the addition of MRA to ACE-I and/or ARB compared with ACE-I and/or ARB alone. For participant numbers see Table 1

There was a significant change in systolic blood pressure (SBP) at final visit (−5.7 mmHg) and in the nine trials where change from baseline (−3.3 mmHg) was available, with addition of MRA to ACE-I and/or ARB in comparison to ACE-I and/or ARB alone (Fig. 2a). Addition of MRA to ACE-I and/or ARB also led to a significant change in end of trial diastolic blood pressure (DBP) (−1.7 mmHg) and change from baseline to final visit (−2.8 mmHg) in comparison to ACE-I and/or ARB alone (Table 2).

**Table 2:** Effect of addition of MRA on final visit blood pressure

Variable	Measurement	No. of study groups	No. patients in intervention	No. patients in placebo/control	Effect size (95 % CI)	I^2^ (*p* value)
Systolic BP (mmHg)	Change from baseline	9	260	266	−3.30 (−5.56, −1.04)	40.0 % (0.101)
	Final visit	16	666	659	−5.69 (−9.04, −2.34)	81.8 % (0.000)
Diastolic BP (mmHg)	Change from baseline	9	260	266	−2.84 (−3.35, −2.33)	0.0 % (0.799)
	Final visit	16	666	659	−1.73 (−3.37, −0.10)	68.3 % (0.000)

#### Effect of treatment on renal excretory function

There was a small, non-significant increase in end-of-trial serum creatinine (3.8 μmol/L) with addition of MRA to ACE-I and/or ARB compared to ACE-I and/or ARB alone (Table 3). Addition of MRA to ACE-I and/or ARB led to similar change in eGFR (−2.7 mL/min/1.73 m^2^) and CrCl (−2.5 mL/min) compared to ACE-I and/or ARB alone with little heterogeneity between included study groups respectively (I^2^ = 0 %, *P* = 0.696 and I^2^ = 0 %, *P* = 0.727) (Fig. 2b).

**Table 3:** Effect of addition of MRA on final visit renal function and urinary protein/albumin excretion

Variable	Measurement	No. of study groups	No. patients in intervention	No. patients in placebo/control	Effect size (95 % CI)	I^2^ (*p* value)
Creatinine (μmol/L)	Final visit	16	601	595	3.83 (−2.14, 9.79)	50.4 % (0.011)
Creatinine Clearance (ml/min)	Final visit	6	132	130	−2.51 (−7.05, 2.04)	0.0 % (0.599)
eGFR (ml/min/1.73 m^2^)	Final visit	13	626	617	−2.71 (−4.85, −0.57)	0.0 % (0.727)
GFR (any measure)	Final visit	17	692	682	−3.15 (−5.36, −0.95)	0.0 % (0.790)
Urinary ACR (mg/mmol)	Final visit	7	355	351	−10.91 (−26.15, 4.32)	83.4 % (0.000)
Urinary PCR (g/g creatinine)	Final visit	4	146	150	−0.91 (−1.35, −0.46)	58.4 % (0.065)
24 h urinary albumin excretion (mg/24 h)	Final visit	6	151	155	−332.91 (−624.80, −41.02)	66.5 % (0.011)
	Change from baseline	3	90	94	−292.23 (−422.19, −162.27)	0.0 % (0.606)
24 h urinary protein excretion (g/24 h)	Final visit	2	124	121	−0.41 (−0.90, 0.09)	77.1 % (0.037)

#### Effect of treatment on urinary albumin/protein excretion

In trials reporting ACR, there was a non-significant change (−10.91 mg/mmol creatinine) with addition of MRA to ACE-I and/or ARB (Table 3). Where data for end-of-trial 24-h urinary albumin excretion were available, there was significant change associated with MRA in addition to ACE-I and/or ARB (−332.9 mg/24 h). This was confirmed in the 3 trials reporting change from baseline albumin excretion, (−292.23 [−422.2, −162.3] mg/24 h). In trials reporting PCR, MRA added to ACE-I and/or ARB led to significant change in end-of-trial values (−0.91 g/g creatinine) compared with ACE-I and/or ARB alone (Table 3). Change in 24-h urine protein from baseline to end-of-trial was available from two trials and showed a trend towards reduction (−0.41 g/24 h) (Table 3) [34, 41].

Analysis of absolute values may be misleading with non-parametric data; therefore we assessed relative change from baseline in urinary protein/albumin excretion where these data were available. There was a significant reduction in urinary protein/albumin excretion with MRA analysed using the 5 trials which reported percentage change from baseline and where SD were available (Fig. 2c); and also when analysed as percentage change in any measure of urinary protein/albumin excretion. Using difference in means analysis in order to include data from all 19 trials, addition of MRA to ACE-I and/or ARB led to weighted mean reduction of 38.7 % (weighted SD 21.5 %) in any measure of protein/albumin excretion. The pattern of association between reduction in SBP and percentage reduction in urinary protein/albumin excretion is shown in Additional file 1: Figure S1.

#### Effect of treatment on potassium

Addition of MRA to ACE-I and/or ARB led to a moderate increase from baseline potassium compared to ACE-I and/or ARB alone both at end-of-trial visit (0.19 mmol/L [95 % CI 0.07–0.31]; 16 trials; *n* = 1356; I^2^ = 83.8 %) and analysed as change from baseline to end-of-trial (0.19 mmol/L [95 % CI 0.12–0.27]; 8 trials; *n* = 478; I^2^ = 0 %. Baseline serum creatinine had no impact on difference in end of trial potassium (*p* = 0.15) or risk of hyperkalaemia (*p* = 0.21).

**Fig. 3:**
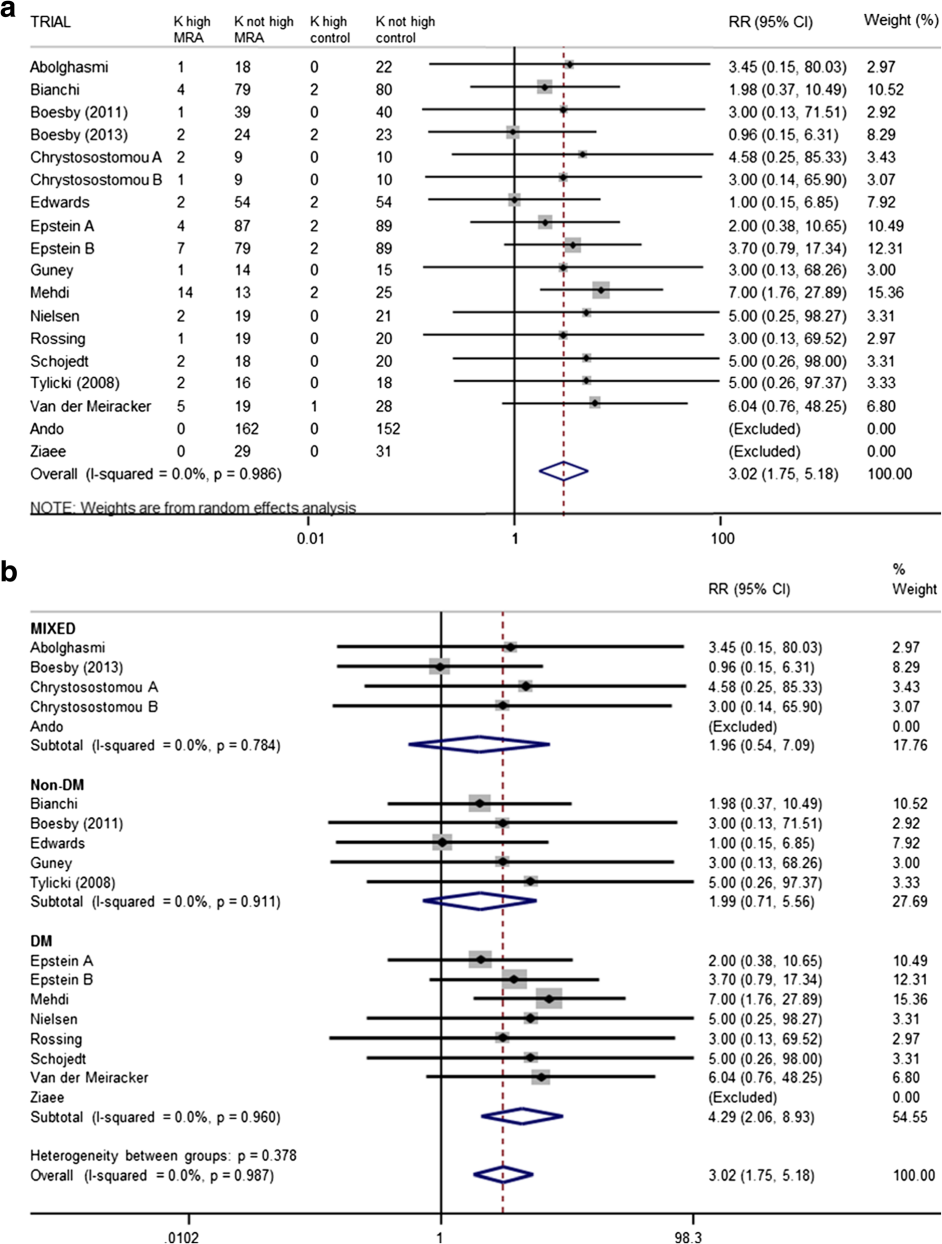
**a** Relative risk of developing hyperkalaemia above the predefined study limit with the addition of MRA to ACE-I and/or ARB compared to ACE-I and/or ARB alone. For participant numbers see Table 1. **b** Relative risk of developing hyperkalaemia above the predefined study limit with the addition of MRA to ACE-I and/or ARB compared to ACE-I and/or ARB alone based on aetiology of CKD (*DM* diabetes mellitus) included in trial. For participant numbers see Table 1

MRA was associated with threefold increased risk of hyperkalaemia above the predefined trial limit (Fig. 3a). Diabetic CKD patients were not at greater risk of developing hyperkalaemia than patients with CKD of alternative aetiology (*p* = 0.38) (Fig. 3b). Number needed to harm for 1 year of treatment, calculated from trials reporting at least one case of hyperkalaemia, was 10 (95 % CI 5–27). Relative risk of being withdrawn from active treatment due to hyperkalaemia was increased (RR 3.21, 95 % CI 1.19, 8.71), equating to number needed to harm over 1 year of 23 (95 % CI 7–267) in trials where at least one patient stopped therapy.

#### Hard clinical endpoints

Included studies were not adequately powered for analysis of hard clinical outcomes. No studies reported participants commencing RRT during the course of the trial. One study reported a participant death (cause unknown) in the treatment group [28] but there were no deaths reported from the remaining 18 studies. Cardiovascular morbidity was reported by two groups [22, 28]. In one study, one patient in the treatment group developed atrial fibrillation and one in the control group suffered a cerebrovascular accident (CVA) [28]. Mehdi et al. reported occurrence of two CVA, two hospitalisations for heart failure, one myocardial infarction and one coronary artery bypass graft in the treatment group; whilst one subject in the placebo group suffered a CVA (Additional file 1: Table S4) [22]. A significant reduction in mortality with addition of MRA was seen in dialysis studies (Additional file 1: Figure S2).

#### Heterogeneity

Heterogeneity was considerable for analyses of most outcomes (SBP, end-of-trial DBP, serum creatinine, urinary ACR and PCR, end-of-trial 24 h urinary albumin). However, heterogeneity was limited for other outcomes (Tables 2 and 3).

#### Publication bias

**Fig. 4:**
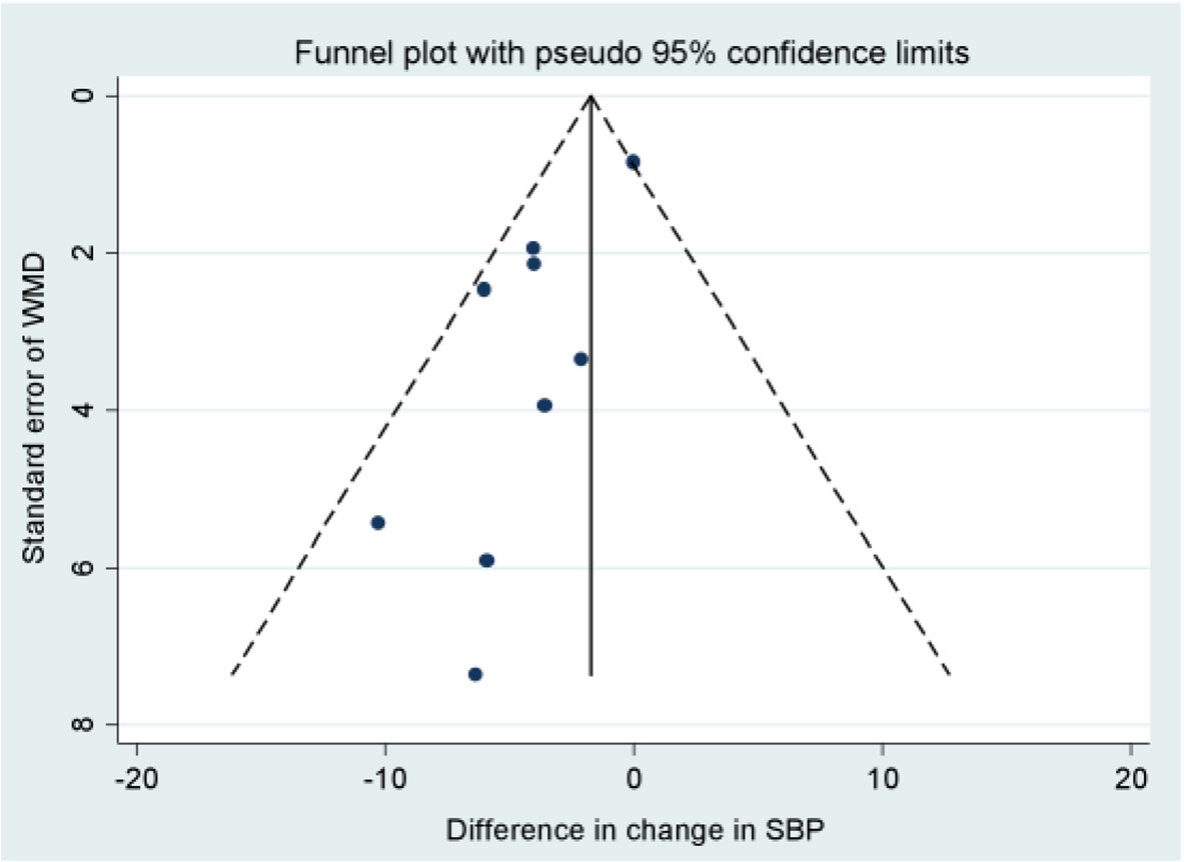
Funnel plot (pseudo 95 % confidence limits) demonstrating some evidence of publication bias for SBP (Egger test *p* = 0.08)

There was some suggestion of publication bias for SBP, evidenced by funnel plot (Fig. 4) and Egger test (*p* = 0.08), but not for end of study GFR or hyperkalaemia (Additional file 1: Figures S3 and S4).

## Discussion

The beneficial impact of RAS blockade with ACE-I and ARB in both diabetic and non-diabetic CKD is well established [8–10, 13]. However, studies of combination therapy suggest harm in particular because of increased risk of hyperkalaemia and/or need for dialysis, and this treatment strategy is therefore generally avoided [44, 45]. Accumulating evidence suggests an independent role for aldosterone in development and progression of CV and renal disease [46–49]. Recent trials included in this analysis demonstrate beneficial effects on BP and urinary protein excretion in CKD with addition of MRA to ACE-I and ARB therapy. These benefits may however be offset by increased risk of hyperkalaemia or decline in renal function.

This meta-analysis confirms that MRA use in combination with ACE-I and/or ARB is associated with significant reductions in BP and end-of-treatment protein/albumin excretion, while conferring a small and quantifiable increased risk of hyperkalaemia. These findings are in keeping with previous published meta-analyses [15, 16, 20]. By using additional unpublished summary results from a number of authors, we ensure that our protein/albumin excretion data are more comprehensive and hence more accurate. Through excluding studies where additional therapy was combined with MRA, we are confident that we report purely the effect of MRA on outcomes. Furthermore, we report clinically relevant risk of hyperkalaemia as relative risk and number needed to harm.

Many studies show strong independent associations between albuminuria and development of ESRD, translating into its widespread use as a surrogate outcome in CKD trials. A recent meta-analysis of over 78 000 patients confirmed that 30 % reduction in albuminuria confers 23.7 % reduction in risk of progression to ESRD, irrespective of drug class [50]. We demonstrate that addition of MRA to ACE-I and/or ARB achieves 40 % reduction in measures of protein/albumin excretion. This should translate to greater benefits in reduction in risk of ESRD and potentially in CV disease [51]. It is possible that reduction in protein/albumin excretion seen with MRA treatment is not entirely BP dependent (Additional file 1: Figure S1). BP independent effects of MRA on proteinuria are difficult to determine in the presence of substantial BP lowering effects with MRA seen across the trials.

Follow-up period of included trials was <1 year and mean baseline eGFR was >35 ml/min/1.73 m^2^ in all studies, therefore impact of addition of MRA to RAS blockade on longer-term renal outcomes or mortality in the later stages of CKD cannot be evaluated. Establishing efficacy of MRA treatment on morbidity and mortality in CKD requires longer follow up and larger sample size. One ongoing study may address this [52]. None of the studies included were powered to detect differences in hard renal end-points, CV morbidity or mortality. We cannot draw conclusions regarding longer-term safety and efficacy of combination MRA and/or ARB, despite postulated CV benefits of reducing protein/albumin excretion [53]. This is in keeping with a meta-analysis published last year [20].

Hyperkalaemia is an inherent risk associated with using MRA combined with RAS blockers and a common occurrence in studies of dual RAS blockade. Data from the ONTARGET study showed that combination ACE–I and ARB increased risk of dialysis, doubling of creatinine or death and combination treatment is not recommended in clinical guidelines [44]. We show an average potassium increase of 0.19 mmol/L at end of treatment (similar to previous data [15, 16]) and three times greater risk of hyperkalaemia in patients receiving MRA in addition to ACE-I and/or ARB. It is essential to consider that this does not necessarily equate to risk of developing clinically significant hyperkalaemia requiring treatment, rather of developing a serum potassium level above the predefined study upper limit, which in many trials was 5.5–6 mmol/L. Although few patients were withdrawn due to hyperkalaemia, many trials reported no withdrawals and only one study reported a single incidence of hyperkalaemia requiring hospital admission for treatment. The implications of these findings are unclear but the small increase in potassium suggests that if patients with high-normal baseline potassium values are excluded from treatment, the risk of dangerous hyperkalaemia may in fact be low. Hyperkalaemia has been associated with risk of renal events in patients with diabetes and nephropathy [54]. Our analysis did not demonstrate diabetic CKD to be at greater risk of developing hyperkalaemia than non-diabetic CKD.

Patients prescribed MRA require regular monitoring of potassium. Although serum potassium levels climb with chronic dosing in dual RAS blockade, the pattern is of early increase followed by steady state, not continuous increase [19]. It is conceivable that benefits conferred by 40 % reduction in proteinuria outweigh the risk of small rises in potassium. Recent data suggest that nonsteroidal mineralocorticoid receptor antagonists may carry a reduced risk of side-effects, and these agents have shown promise for reduction of albuminuria in diabetic CKD [55]. In addition, recent literature highlights the potential for use of potassium binders in higher risk patients [56]. As well as addressing safety, evaluation of economic effects of increased monitoring of potassium compared reduction in CV events or progression of CKD is required.

Our study includes independent systematic searching, data extraction and assessment of study quality by two independent reviewers based on a pre-specified strategy. In addition, many authors provided supplemental summary data thereby enabling more comprehensive analysis. Furthermore, we obtained data from 3 studies which were not included in the previous meta-analyses [28, 30, 34], resulting in analysis of data from a higher total number of patients (1646 vs 1549). This meta-analysis has several limitations. The majority of studies included patients with CKD stages 1–3 and therefore results may not be readily transferrable to populations with more advanced renal disease. There is a lack of long-term follow up data on clinical endpoints such as mortality and CKD progression. Studies included were small and powered to detect differences in surrogate endpoints. Seven included studies had crossover design [26, 29, 31, 39–41, 43] and reporting of methodology was variable. Adequate assessment of trial quality was not possible in all cases. There was significant heterogeneity in measurement of protein/albumin excretion. Authors reported ACR, PCR, 24 h albuminuria/proteinuria or combinations of these. Whilst the results demonstrate a significant reduction in protein or albumin excretion, this highlights that standardisation of measurements would allow consistent analysis, particularly if albuminuria (or equivalent) is used reliably as a surrogate for progression to ESRD [57].

## Conclusion

In conclusion, in patients with CKD, with persistent proteinuria despite RAS inhibition, addition of MRA represents a promising treatment strategy for reducing BP and proteinuria with a quantifiable risk of hyperkalaemia. Appropriately designed larger studies with longer-term follow up are needed to address if MRA added to conventional RAS blockade reduces the risk of CV disease and need for RRT.

## Additional file

Additional file 1: Table S1. Assessment of bias. **Table S2.** Baseline characteristics for individual study groups. **Table S3.** Comparison of baseline data in meta-analyses. **Table S4.** Events reported in trials analysed CVA- cerebrovascular accident; MI- myocardial infarction; CABG- coronary artery bypass graft. **Figure S1.** Plot of percentage reduction in proteinuria/albuminuria (any measure) SBP (mmHg) at final visit across all studies. Each study is represented by a single circle, scaled to number of participants in the study. **Figure S2.** Effect of addition of MRA on all-cause mortality in RRT studies. **Figure S3.** Funnel plot (pseudo 95 % confidence limits) showing no evidence of publication bias for GFR (Egger test *p* = 0.89). **Figure S4.** Funnel plot (pseudo 95 % confidence limits) showing no evidence of publication bias for hyperkalaemia (Egger test *p* = 0.81). **Appendix 1.** Search strategy. **Appendix 2.** Sample data extraction form. (DOCX 192 kb)
