# Supplementary material for: Effect of mineralocorticoid receptor antagonists on proteinuria and progression of chronic kidney disease: a systematic review and meta-analysis
Source: BMC Nephrol. 2016 Sep 8;17(1):127. doi: 10.1186/s12882-016-0337-0 (PMC5015203; doi:10.1186/s12882-016-0337-0)
Supplement: Additional file 1: Table S1. — Assessment of bias. Table S2. Baseline characteristics for individual study groups. Table S3. Comparison of baseline data in meta-analyses. Table S4. Events reported in trials analysed CVA- cerebrovascular accident; MI- myocardial infarction; CABG- coronary artery bypass graft. Figure S1. Plot of percentage reduction in proteinuria/albuminuria (any measure) SBP (mmHg) at final visit across all studies. Each study is represented by a single circle, scaled to number of participants in the study. Figure S2. Effect of addition of MRA on all-cause mortality in RRT studies. Figure S3. Funnel plot (pseudo 95 % confidence limits) showing no evidence of publication bias for GFR (Egger test p = 0.89). Figure S4. Funnel plot (pseudo 95 % confidence limits) showing no evidence of publication bias for hyperkalaemia (Egger test p = 0.81). Appendix 1. Search strategy. Appendix 2. Sample data extraction form. (DOCX 192 kb) [file 12882_2016_337_MOESM1_ESM.docx]

**Table S1: Assessment of bias**

| **Trial** | **Random sequence generation (selection bias)** | **Allocation concealment (selection bias)** | **Blinding of participants**  **(performance bias)** | **Blinding of personnel (performance bias)** | **Blinding of outcome assessment (detection bias)** | **Incomplete outcome data (attrition bias)** | **Selective reporting (reporting bias)** | **Intention to treat analysis** |
| --- | --- | --- | --- | --- | --- | --- | --- | --- |
| **Abolghasmi**  **2011** | **Unclear risk**  (don’t state how participants were randomised) | **Unclear risk** | **Low risk**  (double-blind) | **Low risk**  (double blind) | **Low risk**  (not stated in paper but unlikely that biochemical outcomes would be influenced) | **Unclear risk**  (don’t report whether all patients completed study) | **Low risk**  (protocol not available but all prespecified outcome measures reported) | **Not performed** |
| **Ando**  **2014** | **Low risk**  (computer generated list) | **Low risk**  (list created by central statistician, block size concealed to all investigators) | **Low risk**  (double blind) | **Low risk**  (double blind) | **Low risk**  (data collection and management personnel and statisticians all blinded for duration) | **Low risk**  (losses to follow up disclosed – 18, should not affect results as predicted drop-out rate in sample size calculation was 10%) | **Low risk**  (All prespecified outcomes were reported) | **Yes** |
| **Bianchi**  **2006** | **Low risk**  (computer generated list) | **Unclear risk**  (further description of allocation not included) | **High risk**  (open label) | **High risk**  (open label) | **High risk**  (open label) | **Low risk**  (drop-outs disclosed) | **Low risk**  (all prespecified outcomes reported, most drop outs from high risk group were as a results of K+ but this was disclosed in paper) | **Not performed** |
| **Boesby**  **2011** | **Unclear risk**  (“investigator drew sealed opaque envelopes” but no detail on how randomisation list created) | **Low risk**  (sealed, opaque envelopes) | **High risk**  (open label XO) | **High risk**  (open label XO) | **High risk**  (open label XO) | **Low risk**  (drop-outs disclosed) | **Low risk**  (all prespecified outcomes reported) | **Modified**  (drop outs after randomisation still included in final analysis) |
| **Boesby**  **2013** | **Unclear risk**  (“randomisation done by GCP-unit” but no detail on how) | **Unclear risk**  (not documented) | **High risk**  (open label) | **High risk**  (open label) | **High risk**  (open label) | **Low risk**  (drop-outs disclosed, groups still equal) | **Low risk**  (all prespecified outcomes reported) | **Not performed** |
| **Chrystosostomou**  **2006** | **Unclear risk**  (“randomisation done by clinical trial pharmacists not involved in study”) | **Low risk**  (simple randomisation) | **Low risk**  (double blind) | **Low risk**  (double blind) | **Low risk**  (unblinded after 3 months but unlikely to have influenced outcome measures) | **Low risk**  drop outs disclosed but only 1 so unlikely to affect outcome) | **Low risk**  (all prespecified outcomes reported) | **Yes** |
| **Edwards**  **2009** | **Unclear risk**  (no detail on how sequence generated) | **Unclear risk**  (no detail on how allocation performed) | **Low risk**  (double blind) | **Low risk**  (double blind) | **Low risk**  (not stated in paper but unlikely that biochemical outcomes would be influenced) | **Low risk**  (drop outs disclosed) | **Low risk**  (all prespecified outcomes reported) | **Not performed** |
| **Epstein**  **2006** | **Unclear risk**  (no detail on how randomisation performed) | **Unclear risk**  (no detail given) | **Low risk**  (double blind) | **Low risk**  (double blind) | **Low risk**  (not stated in paper but unlikely that biochemical outcomes would be influenced) | **Low risk**  (drop outs fully disclosed) | **Low risk**  (all prespecified outcomes reported) | **Not performed** |
| **Guney**  **2009** | **Unclear risk**  (no detail on how randomisation performed) | **Unclear risk**  (no detail given) | **High risk**  (not blinded) | **High risk**  (not blinded) | **High risk**  (not blinded) | **Low risk**  (drop outs fully disclosed) | **Low risk**  (all prespecified outcomes reported) | **Not performed** |
| **Mehdi**  **2009** | **Low risk**  (computer randomisation, by diabetes type) | **Low risk**  (performed by staff at investigational study drug unit) | **Low risk**  (double blind) | **Low risk**  (double blind) | **Low risk**  (not stated in paper but unlikely that biochemical outcomes would be influenced) | **Low risk**  (drop outs disclosed but only 1 so unlikely to affect outcome) | **Low risk**  (all prespecified outcomes reported) | **Yes** |
| **Nielsen**  **2012** | **Low risk**  (computer generated randomisation) | **Low risk**  (unknown block size and frequency) | **Low risk**  (double blind) | **Low risk**  (double blind) | **Low risk**  (not stated in paper but unlikely that biochemical outcomes would be influenced) | **Unclear risk**  (don’t report whether all patients completed study) | **Low risk**  (all prespecified outcomes reported) | **Unable to comment - ?assume all participants completed study** |
| **Rossing**  **2005** | **Low risk**  (computer generated randomisation) | **Low risk**  (sealed envelopes) | **Low risk**  (double blind) | **Low risk**  (double blind) | **Low risk**  (“code not broken until all data entered into a database which was locked for editing”) | **Low risk**  (drop outs disclosed but only 1 so unlikely to affect outcome) | **Low risk**  (all prespecified outcomes reported) | **Not performed** |
| **Saklayen**  **2008** | **Unclear risk**  (“sequence generated randomly by a clinical trials pharmacist”) | **Unclear risk**  (no detail given) | **Low risk**  (double blind) | **Low risk**  (double blind) | **Low risk**  (“investigators blinded until code was broken at end of study”) | **Low risk**  (drop outs fully disclosed) | **Unclear risk**  (intended outcome measures not stated in methods section) | **Not performed** |
| **Schojedt**  **2005** | **Low risk**  (computer generated) | **Low risk**  (concealed with computer generated envelopes) | **Low risk**  (double blind) | **Low risk**  (double blind) | **Low risk**  (“code not broken until all data entered into a database which was locked for editing”) | **Low risk**  (drop outs fully disclosed) | **Low risk**  (all prespecified outcomes reported) | **Not performed** |
| **Tylicki**  **2008** | **Low risk**  (computer generated) | **Low risk**  (“independent of study personnel”) | **High risk**  (not blinded) | **High risk**  (not blinded) | **High risk**  (not blinded) | **Low risk**  (no drop outs) | **Low risk**  (all prespecified outcomes reported) | **Not performed/required** |
| **Tylicki**  **2012** | **Low risk**  (computer generated) | **Unclear risk**  (no detail given) | **Low risk**  (double blind) | **Low risk**  (double blind) | **Low risk**  (not stated in paper but unlikely that biochemical outcomes would be influenced) | **Low risk**  (no drop outs) | **Low risk**  (all prespecified outcomes reported) | **Not performed/required** |
| **Van der Meiracker**  **2006** | **Low risk**  (computer generated) | **Unclear risk**  (no detail given) | **Low risk**  (double blind) | **Low risk**  (double blind) | **Low risk**  (not stated in paper but unlikely that biochemical outcomes would be influenced) | **Low risk**  (drop outs fully disclosed) | **Unclear risk**  (intended outcome measures not stated in methods section) | **Not performed** |
| **Wang**  **2013** | **Unclear risk**  (minimal detail on method of randomisation) | **High risk**  (open randomisation) | **High risk**  (not blinded) | **High risk**  (not blinded) | **High risk**  (not blinded) | **Low risk**  (drop outs fully disclosed) | **Low risk**  (all prespecified outcomes reported) | **Not performed** |
| **Ziaee**  **2013** | **Unclear risk**  (no detail on how randomisation performed) | **Unclear risk**  (no detail on how randomisation performed) | **High risk**  (not blinded) | **High risk**  (not blinded) | **High risk**  (not blinded) | **Unclear risk**  (don’t report whether all patients completed study) | **Unclear risk**  (intended outcome measures not stated in methods section) | **Not performed** |

**Table S2: Baseline characteristics for individual study groups.**

| **Study** | **Number of patients** | | **Age**  **(years)** | | **Gender**  **(No. (%) female)** | | **eGFR or Creatinine Clearance**  **(ml/min/1.73m^2^)** | | **Urinary protein/albumin measurement**  **(units)** | **MRA** | **Control** |
| --- | --- | --- | --- | --- | --- | --- | --- | --- | --- | --- | --- |
|  | **MRA** | **Control** | **MRA** | **Control** | **MRA** | **Control** | **MRA** | **Control** |  |  |  |
| **Abolghasmi**  **2011** | 19 | 22 | 49(±13) | 50(±10) | 9(47) | 10(45) | - | - | - | - | - |
| **Ando**  **2014** | 162 | 152 | 59(±13) | 59(±14) | 48(30) | 52(34) | 68(±14) | 69(±14) | ACR  (mg/g) | 163(±148) | 157(±134) |
| **Bianchi**  **2006** | 83 | 82 | 55(±11) | 54(±11) | 27(33) | 32(39) | 62(±22) | 62(±19) | PCR  (g/g) | 2.1(±0.7) | 2(±0.6) |
| **Boesby**  **2011** | 40 | 40 | 45(21-71) | 45(21-71) | 13(33) | 13(33) | 59(±26) | 59(±26) | ACR  (mg/mmol) | 129(±80) | 129(±80) |
| **Boesby**  **2013** | 26 | 25 | 58(±13) | 59(±13) | 7(27) | 6(24) | 36(±10) | 35(±13) | ACR  (mg/mmol) | 51(±56) | 88(±155) |
| **Chrystosostomou A**  **2006** | 11 | 10 | 56(±15) | 56(±9) | 5(45) | 2(20) | *57[41.3-73.9]*** | *68[41.7-94.3]*** | 24hr urinary protein  (g/24hr) | 3.1(±1.9) | 2.5(±1.8) |
| **Chrystosostomou B**  **2006** | 10 | 10 | 66(±9) | 59(±10) | 3(30) | 3(30) | *59[47.3-71.4]*** | *82[48.7-114.4]*** | 24hr urinary protein  (g/24hr) | 2.2(±1.4) | 2.6(±1.6) |
| **Edwards**  **2009** | 56 | 56 | 54(±12) | 53(±12) | 22(39) | 20(36) | 49(±12) | 53(±11) | ACR  (mg/mmol) | 18(±49) | 8.2(±48) |
| **Epstein A**  **2006** | 91 | 91 | 58(52,66) | 60(53,66) | 31(34) | 41(45) | 73(61,84) | 74(61,82) | ACR  (mg/g) | 422(154,856) | 280(105,762) |
| **Epstein B**  **2006** | 86 | 91 | 58(53,66) | 60(53,66) | 30(35) | 41(45) | 75(63,86) | 74(61,82) | ACR  (mg/g) | 240(91,578) | 280(105,762) |
| **Guney**  **2009** | 12 | 12 | 46(±11) | 39(±13) | 3(25) | 4(33) | 63(±23) | 56(±36) | PCR  (mg/mg) | 2.4(±4.9) | 1.5(±2.3) |
| **Mehdi**  **2009** | 27 | 27 | 52(±9) | 49(±9) | 14(52) | 15(56) | *51[40-66]†* | *73[60-89]†* | ACR  (mg/g) | 1094[758-1579]† | 917[633-1329]† |
| **Nielsen**  **2012** | 21 | 21 | 58(±10) | 58(±10) | 7(33) | 7(33) | - | - | 24hr urinary albumin  (mg/24hr) | 96(±65) | 95(±65) |
| **Rossing**  **2005** | 20 | 20 | 58(±10) | 58(±10) | 3(15) | 3(15) | - | - | 24hr urinary albumin  (mg/24hr) | 2068(±1988) | 2068(±1988) |
| **Saklayen**  **2008** | 24 | 24 | 65(±10) | 65(±10) | 0(0) | 0(0) | 62(±23) | 54(±20) | PCR | 1.8(±1.8) | 1.2(±1.1) |
| **Schojedt**  **2005** | 20 | 20 | 45(±7) | 45(±7) | 5(25) | 5(25) | - | - | 24hr urinary albumin  (mg/24hr) | 1307(±1122) | 1307(±1122) |
| **Tylicki**  **2008** | 18 | 18 | 42(±8) | 42(±8) | 7(39) | 7(39) | 108[93-141]† | 108[93-141]† | 24hr urinary protein  (g/24hr) | 0.97(0.8)* | 0.97(0.8)* |
| **Tylicki**  **2012** | 18 | 18 | 39(±11) | 39(±11) | 4(22) | 4(22) | *94(8)** | *94(8)** | - | - | - |
| **Van der Meiracker**  **2006** | 24 | 29 | 55(38,78)‡ | 55(29,75)‡ | 7(29) | 12(41) | 93(±45) | 66(±35) | ACR  (mg/mmol) | 74(±59) | 129(±118) |
| **Wang**  **2013** | 106 | 102 | 34(±8) | 35(±10) | 45(42) | 45(44) | 66(±22) | 67(±24) | 24hr urinary protein  (g/24hr) | 1.9(±0.7) | 1.9(±0.8) |
| **Ziaee**  **2013** | 29 | 31 | 53(±5) | 53(±5) | 12(41) | 11(35) | 80(±18) | 83(±19) | ACR  (mg/mmol) | 126(±69) | 119(±67) |

Table S2: Baseline characteristics for individual study groups. Data expressed as mean (SD), median (IQR), median (range), *mean (SEM), ** mean (95% CI), † geometric mean [95% CI], ‡ geometric mean (IQR). MRA (mineralocorticoid receptor antagonist), ACR (albumin creatinine ratio), PCR (protein creatinine ratio), eGFR (estimated glomerular filtration rate).

**Table S3: Comparison of baseline data in meta-analyses**

| **Variable** | **Units** | **No. of trials** | **No. patients in treatment** | **No. patients in placebo/control** | **Effect size (95% CI)** | **I^2^ (p value)** |
| --- | --- | --- | --- | --- | --- | --- |
| Systolic BP | mmHg | 17 | 693 | 686 | 0.46 (-0.75, 1.66) | 0.0% (0.981) |
| Diastolic BP | mmHg | 17 | 693 | 686 | 0.63 (-0.18, 1.43) | 0.0% (0.739) |
| Serum Potassium | mmol/L | 17 | 708 | 702 | -0.01 (-0.05, 0.04) | 0.0% (0.992) |
| Creatinine | µmol/L | 18 | 646 | 640 | -0.20 (-4.50, 4.10) | 25.1% (0.160) |
| Estimated GFR | ml/min/1.73m^2^ | 9 | 459 | 454 | -0.10 (-3.13, 2.94) | 35.1% (0.137) |
| Creatinine Clearance | ml/min | 6 | 132 | 130 | -3.30 (-9.64, 3.04) | 16.5% (0.307) |
| Urinary protein creatinine ratio | mg/mmol | 4 | 146 | 150 | 0.06 (-0.68, 0.20) | 19.6% (0.292) |
| Urinary albumin creatinine ratio | g/g creatinine | 7 | 364 | 360 | 3.44 (-8.82, 15.71) | 64.8% (0.009) |
| 24 hr urinary protein excretion | g/24 hours | 4 | 145 | 140 | 0.04 (-0.14, 0.22) | 0.0% (0.815) |
| 24 hr urinary albumin excretion | mg/24 hours | 6 | 151 | 155 | -3.26 (-42.14, 35.62) | 0.0% (0.716) |

**Table S4**. Events reported in trials analysed CVA- cerebrovascular accident; MI- myocardial infarction; CABG- coronary artery bypass graft

| **Trial** | **Event** | **Events in control arm** | **Events in MRA arm** |
| --- | --- | --- | --- |
| Ando 2014 |  |  |  |
|  | Death | 0 | 1 (0.6%) |
|  | Atrial fibrillation | 0 | 1 (0.6%) |
|  | CVA | 1 (0.7%) | 0 |
| Medhi 2009 | Heart failure | 2 (3.8%) | 0 |
|  | CVA | 2 (3.8%) | 1 (3.7%) |
|  | MI | 1 (1.9%) | 0 |
|  | CABG | 1 (1.9%) | 0 |

**Figure S1:** Plot of percentage reduction in proteinuria/albuminuria (any measure) SBP (mmHg) at final visit across all studies. Each study is represented by a single circle, scaled to number of participants in the study.

**Figure S2:** Effect of addition of MRA on all-cause mortality in RRT studies^1-6^.


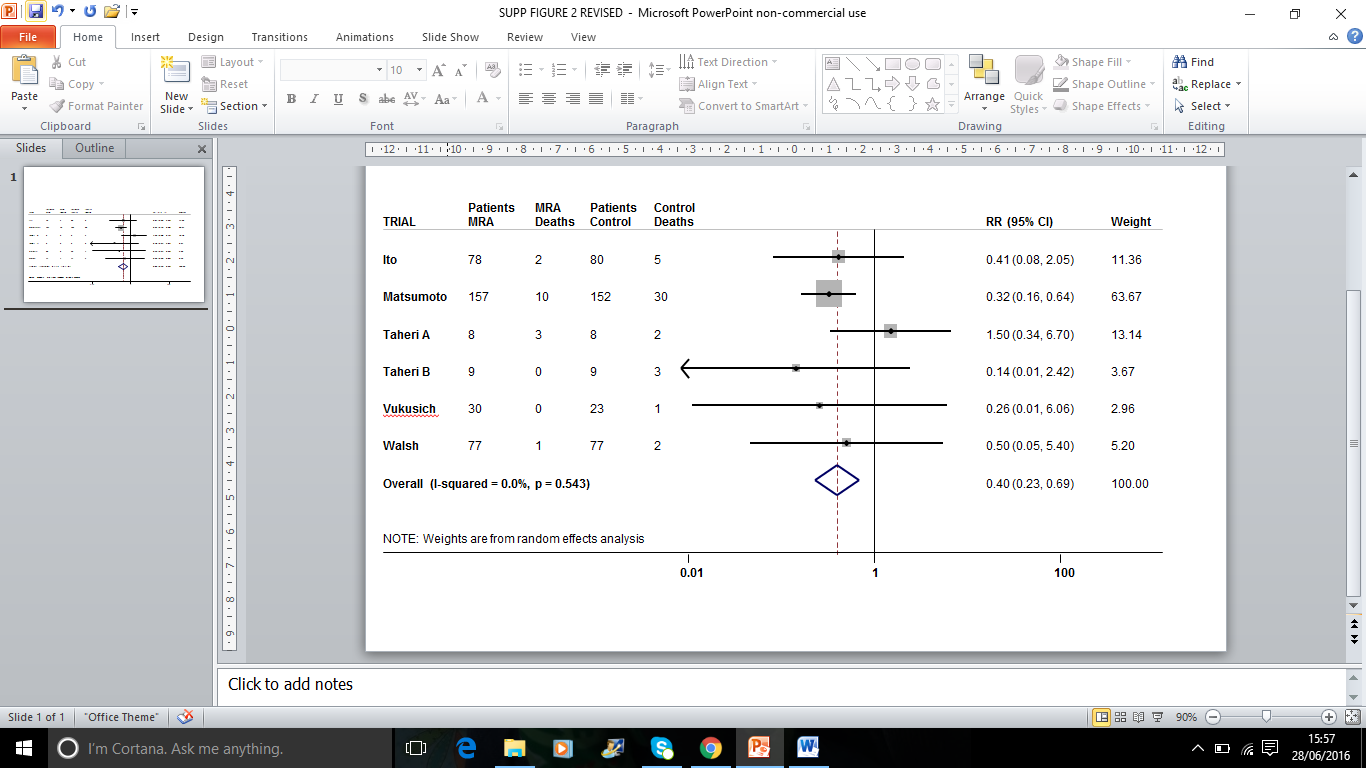


**Figure S3.** Funnel plot (pseudo 95% confidence limits) showing no evidence of publication bias for GFR (Egger test p=0.89).

**Figure S4.** Funnel plot (pseudo 95% confidence limits) showing no evidence of publication bias for hyperkalaemia (Egger test p=0.81).

**REFERENCES For Figure S2**

1. Ito Y, Mizuno M, Suzuki Y, et al. Long-term effects of spironolactone in peritoneal dialysis patients. J Am Soc Nephrol 2014; 25:1094-102.

2. Matsumoto Y, Mori Y, Kageyama S, et al. Spironolactone reduces cardiovascular and cerebrovascular morbidity and mortality in hemodialysis patients. J Am Coll Cardiol 2014; 63:528-36.

3. Taheri S, Mortazavi M, Shahidi S, et al. Spironolactone in chronic hemodialysis patients improves cardiac function. Saudi J Kidney Dis Transpl 2009; 20:392-7.

4. Taheri S, Mortazavi M, Pourmoghadas A, et al. A prospective double-blind randomized placebo-controlled clinical trial to evaluate the safety and efficacy of spironolactone in patients with advanced congestive heart failure on continuous ambulatory peritoneal dialysis. Saudi J Kidney Dis Transpl 2012; 23:507-12.

5. Vukusich A, Kunstmann S, Varela C, et al. A randomized, double-blind, placebo-controlled trial of spironolactone on carotid intima-media thickness in nondiabetic hemodialysis patients. Clin J Am Soc Nephrol 2010; 5:1380-7.

6. Walsh M, Manns B, Garg AX, et al. The Safety of Eplerenone in Hemodialysis Patients: A Noninferiority Randomized Controlled Trial. Clin J Am Soc Nephrol 2015.

**Appendix 1: Search strategy.**

We searched the following databases for relevant clinical studies.

1. EMBASE (1947 – December 2014)
2. Aldo antagonist*.mp
3. Mineralocorticoid receptor blocker*.mp
4. Mineralocorticoid receptor antagonist*.mp
5. Mineralocorticoid antagonist*.mp
6. Spironolactone*.mp
7. Potassium Canrenoate*.mp
8. Canrenone*.mp
9. Aldadiene Potassium*.mp
10. Aldadiene*.mp
11. SC-9376*.mp
12. SC-9420*.mp
13. SC-14266*.mp
14. Eplerenone*.mp
15. Aldactone*.mp
16. Novospiroton*.mp
17. Spiractin*.mp
18. Soldactone*.mp
19. Soludactone*.mp
20. Practon*.mp
21. Phanurane*.mp
22. Luvion*.mp
23. Contaren*.mp
24. Eplecard*.mp
25. Epleran*.mp
26. Eptus*.mp
27. Planep*.mp
28. Chronic renal disease*.mp
29. Chronic renal failure*.mp
30. Chronic kidney insufficiency*.mp
31. Chronic renal insufficiency*.mp
32. Chronic kidney disease*.mp
33. Chronic kidney failure*.mp
34. Proteinuria*.mp
35. Albuminuria*.mp
36. Renal replacement therapy*.mp
37. Dialysis*.mp
38. Haemodialysis*.mp
39. Peritoneal dialysis*.mp
40. Kidney transplant*.mp
41. Renal transplant*.mp
42. Kidney transplantation*.mp
43. Renal transplantation*.mp

**SEARCH 1 AND SEARCH 2 = 116**

1. PubMed (1966 – December 2014)
2. MeSH Aldosterone antagonist
3. Canrenoate Potassium (tw)
4. Canrenone$(tw)
5. Aldadiene potassium (tw)
6. Aldadiene(tw)
7. Spironolactone(tw)
8. Eplerenone(tw)
9. Aldactone(tw)
10. Aldosterone antagonist(tw)
11. Practon(tw)
12. Phamurane(tw)
13. Soldactone(tw)
14. Soludactone(tw)
15. Spiractin(tw)
16. Novospiroton(tw)
17. SC-9376(tw)
18. SC-9420(tw)
19. SC-14266(tw)
20. Luvion(tw)
21. Contaren(tw)
22. Eplecard(tw)
23. Epleran(tw)
24. Planep(tw)
25. Mineralocorticoid Receptor Antagonist(tw)
26. Mineralocorticoid Receptor Blocker(tw)
27. Chronic kidney disease(tw)
28. Chronic renal disease(tw)
29. Chronic kidney failure(tw)
30. Chronic renal failure(tw)
31. Chronic kidney insufficiency(tw)
32. Chronic renal insufficiency(tw)
33. MeSH chronic renal failure
34. MeSH chronic kidney failure
35. MeSH chronic kidney insufficiency
36. MeSH chronic renal insufficiency
37. MeSH renal replacement therapy
38. MeSH dialysis peritoneal
39. MeSH dialysis renal
40. MeSH proteinuria
41. MeSH albuminuria
42. MeSH renal transplant
43. MeSH kidney transplant
44. Proteinuria$(tw)
45. albuminuria$(tw)
46. renal replacement therapy$(tw)
47. haemodialysis$(tw)
48. dialysis$(tw)
49. peritoneal dialysis$(tw)
50. renal transplantation$(tw)
51. kidney transplant$(tw)

**SEARCH 1 AND SEARCH 2 = 57**

1. Cochrane (1947 – December 2014)

1) MeSH Aldosterone antagonist

2) Canrenoate Potassium (tw)

3) Canrenone$(tw)

4) Aldadiene potassium (tw)

5) Aldadiene(tw)

6) Spironolactone(tw)

7) Eplerenone(tw)

8) Aldactone(tw)

9) Aldosterone antagonist(tw)

10) Practon(tw)

11) Phamurane(tw)

12) Soldactone(tw)

13) Soludactone(tw)

14) Spiractin(tw)

15) Novospiroton(tw)

16) SC-9376(tw)

17) SC-9420(tw)

18) SC-14266(tw)

19) Luvion(tw)

20) Contaren(tw)

21) Eplecard(tw)

22) Epleran(tw)

23) Planep(tw)

24) Mineralocorticoid Receptor Antagonist(tw)

25) Mineralocorticoid Receptor Blocker(tw)

1) Chronic kidney disease(tw)

2) Chronic renal disease(tw)

3) Chronic kidney failure(tw)

4) Chronic renal failure(tw)

5) Chronic kidney insufficiency(tw)

6) Chronic renal insufficiency(tw)

7) MeSH chronic renal failure

8) MeSH chronic kidney failure

9) MeSH chronic kidney insufficiency

10) MeSH chronic renal insufficiency

11) MeSH renal replacement therapy

12) MeSH dialysis peritoneal

13) MeSH dialysis renal

14) MeSH proteinuria

15) MeSH albuminuria

16) MeSH renal transplant

17) MeSH kidney transplant

18) Proteinuria$(tw)

19) albuminuria$(tw)

20) renal replacement therapy$(tw)

21) haemodialysis$(tw)

22) dialysis$(tw)

23) peritoneal dialysis$(tw)

24) renal transplantation$(tw)

25) kidney transplant$(tw)

**SEARCH 1 AND SEARCH 2 = 126**

**Appendix 2: Sample data extraction form**

Dear colleague, thank you for considering our proposal to join the above meta-analysis. The following is a list of data required to enable us to incorporate data from your trial in to the meta-analysis.

1. Blood Pressure (office measurement)
   1. Mean systolic BP (±SD) at baseline
      1. Treatment group (±SD) ………………………………………….
      2. Control group (±SD) ………………………………………….
   2. Mean diastolic BP (±SD) at baseline
      1. Treatment group (±SD) ………………………………………….
      2. Control group (±SD) ………………………………………….
   3. Mean systolic BP (±SD) at end of study
      1. Treatment group (±SD) ………………………………………….
      2. Control group (±SD) ………………………………………….
   4. Mean diastolic BP (±SD) at end of study
      1. Treatment group (±SD) ………………………………………….
      2. Control group (±SD) ………………………………………….
   5. Mean change (±SD) in systolic BP from baseline
      1. Treatment group (±SD) ………………………………………….
      2. Control group (±SD) ………………………………………….
   6. Mean change (±SD) in diastolic BP from baseline
      1. Treatment group (±SD) ………………………………………….
      2. Control group (±SD) ………………………………………….
2. Renal Function
   1. Mean serum creatinine (±SD) at baseline
      1. Treatment group (±SD) ………………………………………….
      2. Control group (±SD) ………………………………………….
   2. Mean serum creatinine (±SD) at end of study
      1. Treatment group (±SD) ………………………………………….
      2. Control group (±SD) ………………………………………….
   3. Mean change (±SD) in serum creatinine from baseline
      1. Treatment group (±SD) ………………………………………….
      2. Control group (±SD) ………………………………………….
   4. Mean GFR (±SD) at baseline (actual or estimated GFR accepted)
      1. Treatment group (±SD) ………………………………………….
      2. Control group (±SD) ………………………………………….
   5. Mean GFR (±SD) at end of study (actual or estimated GFR accepted)
      1. Treatment group (±SD) ………………………………………….
      2. Control group (±SD) ………………………………………….
   6. Mean change (±SD) in GFR from baseline (actual or estimated GFR accepted)
      1. Treatment group (±SD) ………………………………………….
      2. Control group (±SD) ………………………………………….
3. Renal protein/albumin excretion

Please specify whether proteinuria or albuminuria has been measured (PCR or ACR).

- 1. Mean protein/albumin creatinine ratio (±SD) at baseline
     1. Treatment group (±SD) ………………………………………….
     2. Control group (±SD) ………………………………………….
  2. Mean protein/albumin creatinine ratio (±SD) at end of study
     1. Treatment group (±SD) ………………………………………….
     2. Control group (±SD) ………………………………………….
  3. Mean change in protein/albumin creatinine ratio (±SD) from baseline
     1. Treatment group (±SD) ………………………………………….
     2. Control group (±SD) ………………………………………….
  4. Mean 24 hour protein/albumin excretion (±SD) at baseline
     1. Treatment group (±SD) ………………………………………….
     2. Control group (±SD) ………………………………………….
  5. Mean 24 hour protein/albumin excretion (±SD) at end of study
     1. Treatment group (±SD) ………………………………………….
     2. Control group (±SD) ………………………………………….
  6. Mean change in 24 hour protein/albumin excretion (±SD) from baseline
     1. Treatment group (±SD) ………………………………………….
     2. Control group (±SD) ………………………………………….

1. Serum potassium
   1. Mean serum potassium (±SD) at baseline
      1. Treatment group (±SD) ………………………………………….
      2. Control group (±SD) ………………………………………….
   2. Mean serum potassium (±SD) at end of study
      1. Treatment group (±SD) ………………………………………….
      2. Control group (±SD) ………………………………………….
   3. Mean change in serum potassium (±SD) from baseline
      1. Treatment group (±SD) ………………………………………….
      2. Control group (±SD) ………………………………………….
   4. Number of patients who developed Hyperkalaemia (K+>5.5) after baseline visit
      1. Treatment group (±SD) ………………………………………….
      2. Control group (±SD) ………………………………………….
